# CCN1 Induces Oncostatin M Production in Osteoblasts via Integrin-Dependent Signal Pathways

**DOI:** 10.1371/journal.pone.0106632

**Published:** 2014-09-04

**Authors:** Cheng-Yu Chen, Chen-Ming Su, Yuan-Li Huang, Chun-Hao Tsai, Lih-Jyh Fuh, Chih-Hsin Tang

**Affiliations:** 1 Graduate Institute of Basic Medical Science, China Medical University, Taichung, Taiwan; 2 Department of Biotechnology, College of Health Science, Asia University, Taichung, Taiwan; 3 Department of Medicine and Graduate Institute of Clinical Medical Science, China Medical University, Taichung, Taiwan; 4 Department of Orthopedic Surgery, China Medical University Hospital, Taichung, Taiwan; 5 Department of Prosthodontics, China Medical University Hospital, Taichung, Taiwan; 6 Department of Pharmacology, School of Medicine, China Medical University, Taichung, Taiwan; Chang Gung University, Taiwan

## Abstract

Inflammatory response and articular destruction are common symptoms of osteoarthritis. Cysteine-rich 61 (CCN1 or Cyr61), a secreted protein from the CCN family, is associated with the extracellular matrix involved in many cellular activities like growth and differentiation. Yet the mechanism of CCN1 interacting with arthritic inflammatory response is unclear. This study finds CCN1 increasing expression of oncostatin m (OSM) in human osteoblastic cells. Pretreatment of αvβ3 monoclonal antibody and inhibitors of focal adhesion kinase (FAK), c-Src, phosphatidylinositol 3-kinase (PI3K), and NF-κB inhibited CCN1-induced OSM expression in osteoblastic cells. Stimulation of cells with CCN1 increased phosphorylation of FAK, c-Src, PI3K, and NF-κB via αvβ3 receptor; CCN1 treatment of osteoblasts increased NF-κB-luciferase activity and p65 binding to NF-κB element on OSM promoter. Results indicate CCN1 heightening OSM expression via αvβ3 receptor, FAK, c-Src, PI3K, and NF-κB signal pathway in osteoblastic cells, suggesting CCN1 as a novel target in arthritis treatment.

## Introduction

Arthritis as a systemic inflammatory process comprises osteoarthritis (OA) and rheumatoid arthritis (RA) that leads to joint destruction and extra articular symptoms, with significant effect on morbidity and mortality [1]–[3]. As cartilages impaired or monocytes infiltrated the synovium, proinflammatory cytokines were secreted during development of arthritis that caused synovial hyperplasia, secretion of degradative enzymes, and bone long-term erosion and damage [4], [5]. Previous study showed chemokines released directly or indirectly from subchondral bone that caused bone remodelling and cartilage destruction in arthritis [6]. As cartilage was depreciated in OA pathogenesis, some studies indicated subchondral bone also playing a key role in OA and RA process [7], [8]. Hence, subchondral bone potentially acts in concert as a mechanical environment in response to development of arthritis.

Oncostatin M (OSM), 28-kDa, a cytokine of the interleukin-6 (IL-6) family, is multifunctional (skeletal tissue alteration, bone metabolism, inflammatory disease) and originates from monocytes, macrophages, or T cells within chronic inflammatory process [5], [9], [10]. Studies indicated OSM omnipresent in synovial fluid and serum in OA and RA cases [11]–[13], while resulting in secretion of proinflammatory cytokines: TNF-α, IL-1β, and IL-6 from osteoblasts and synovial cells that degrade cartilage in arthritic joints [14]–[16], hinting OSM’s role in pathogenesis. CCN1, cysteine-rich 61 (Cyr61) attached to CCN family, has multiple effects on physiology or pathology or immunology attributable to its receptor in diverse cell types [17]. It is crucial to mediating cell adhesion and inducing cell proliferation, and it also regulates chronic inflammation, wound healing, and vascular disease [18]–[20]. Genomic studies show CCN1 strongly expressed in collagen-induced arthritis in rodents, suggesting CCN1 inhibitor reduces inflammatory response [21]. CCN1 promotes fibroblast-like synoviocytes proliferation and activates Th17 cells in arthritis pathogenesis [20]. Numerous studies have shown CCN1 binding integrin to activate downstream signal transduction, while binding of αvβ3 triggers cell adhesion and apoptosis, binding of α6β1 induces senescence, and binding of αvβ5 affects migration [4], [18]. These indicate binding of CCN1 and integrins as pivotal in inflammatory arthritis [4], [9].

Past research showed arthritis correlating with osteoclast differentiation, recent study indicates osteoblasts also participating in inflammation process [22], [23], OSM strongly expressed in osteoblasts isolated from femora in arthritics [6], [23]. OSM can regulate arthritis associated with osteoblasts [16], [24]. Effect of CCN1-induced OSM expression in osteoblasts is yet unclarified. This study investigated signal pathway involved CCN1-induced OSM production in human osteoblasts. Results show CCN1 up-regulating OSM expression via αvβ3 receptor FAK/c-Src/PI3K/NF-κB signal pathway, lending insight into CCN1’s therapeutic value against arthritis.

## Materials and Methods

### Materials

Rabbit polyclonal antibody specific to phosphate p-PI3K was obtained from Cell Signaling Technology (Danvers, MA); rabbit polyclonal antibodies specific to αvβ3, p-FAK, FAK, c-Src, PI3K, p-p65, p65, β-actin, and mouse polyclonal antibodies specific to p-c-Src and OSM from Santa Cruz Biotechnology (Santa Cruz, CA). Human recombinant CCN1 was obtained from PeproTech (Rocky Hill, NJ), FAK inhibitor (FAKi) and c-Src inhibitor (PP2), PI3K inhibitors (Wortmannin and Ly294002), NF-κB inhibitors pyrrolidine dithiocarbamate (PDTC) and L-1-tosylamido-2-phenylenylethyl chloromethyl ketone (TPCK) from Sigma-Aldrich (St. Louis, MO). NF-κB luciferase kit was purchased from Stratagene (La Jolla, CA). DMEM, fetal bovine serum (FBS), all other cell culture reagents from Gibco-BRL Life Technologies (Grand Island, NY).

### Cell culture

Human osteoblast-like cell line MG-63 and mouse osteoblast cell line MC3T3-E1 were purchased from American Type Culture Collection (Manassas, VA), cells maintained in DMEM or α-MEM supplemented with 10% heat-inactivated FBS, penicillin (100 U/ml), at 37°C with 5% CO_2_ incubator.

### Western blot

Cellular lysates were derived from prior study [25], proteins resolved by sodium dodecyl sulfate-polyacrylamide gel electrophoresis and then transferred to polyvinyl difluoride membranes (Millipore, MA). Blots were blocked with 4% non-fat milk for 1 h at room temperature, then probed with rabbit anti-human antibodies against OSM, αvβ3, p-FAK, FAK, c-Src, PI3K, or p-p65, and mouse anti-human antibodies against p-c-Src or OSM (1∶1,000) for 1 h at room temperature. After three washes, blots were incubated for 1 h with goat anti-rabbit or anti-mouse peroxidase-conjugated secondary antibody (1∶1,000) at room temperature, visualized by enhanced chemiluminescence with Kodak X-OMAT LS film (Eastman Kodak, NY).

### Real-time quantitative PCR (RT-qPCR) for mRNA analysis

Total RNA was extracted from osteoblasts by a TRIzol kit (MDBio, Taipei). Reverse transcription proceeded with 1 µg of total RNA and oligo (dT) primer. Real-time quantitative PCR (RT-qPCR) analysis used Taqman one-step PCR Master Mix (Applied Biosystems, CA), 100 ng of total cDNA added per 25 µl reaction with sequence-specific primers and Taqman probes. Sequences for target gene primers and +assays carried out in triplicate by StepOnePlus sequence detection system. Cycling conditions entailed 10-min polymerase activation at 95°C followed by 40 cycles at 95°C for 15 s and 60°C for 60 s, threshold set above non-template control background and within linear phase of target gene amplification to rate cycle number at which transcript was detected (denoted C_T_).

### ELISA assay

Human osteoblasts were cultured in 24-well plates reached confluence and were changed to serum-free medium, then treated with CCN1 alone for 24 h or pretreated with pharmacological inhibitors or transfected with specific siRNA, followed by 24 h CCN1 stimulation. Medium was removed and stored at −80°C, OSM therein derived by OSM ELISA kit (R&D Systems, MN), as per manufacturer’s protocol.

### Plasmids and constructs

Plasmid pGL2-Basic (Promega, Madison, Wis.) was used to generate constructs of the OSM promoter. hOSM were designed between *NheI* and *BglII* and amplified by PCR with chemically synthesized oligonucleotides that corresponded to the sense strand 5′-CCCCGCTAGCTGGGAGTGGCTGGTGCAGCA-3′, and the antisense strand 5′-CCCCAGATCTGGGAGCCCTGCAGGCTGGCA-3′. The constructs containing OSM insert was sequenced and verified.

### Transient transfection and reporter gene assay

Human osteoblasts were plated in 12-well dishes. Human osteoblasts were co-transfectedwith 0.8 µg luciferase plasmids and 0.4 µg β-galactosidase expression vector. DNA and Lipofectamine 2000 (Invitrogen, CA) were premixed for 20 min and then applied to the cells. After 24 h, transfection, the cells were incubated with the indicated agents. Cell extracts were prepared and used for measuring the luciferase and β-galactosidase activities.

ON-TARGETplus siRNAs of FAK, c-Src, PI3K, p65 and control were purchased from Dharmacon Research. Transient transfection of siRNAs used DharmaFECT1 transfection reagent, siRNA (100 nM) formulated with DharmaFECT1 transfection reagent according to manufacturer's instruction.

### Chromatin immunoprecipitation (CHIP) assay

Chromatin immunoprecipitation proceeded as detailed earlier [25], DNA pellets immunoprecipitated by anti-p65 purified and extracted by phenol-chloroform, then subjected to PCR resolved by 2% agarose gel electrophoresis and visualized under UV light, 5′-AAACCTGGTCCCCAACTGTC-3′ and 5′-GGCCTCAGAGACCCAC AACT-3′ primers utilized for amplification across OSM promoter region.

### Statistical analysis

Data represent mean ± standard error of mean. Comparisons between samples used Student’s *t* test. Statistical comparisons of more than two groups used one-way analysis of variance with Bonferroni’s *post-hoc* test, *p*<0.05 considered significant.

## Results

### CCN1 induces OSM expression in osteoblasts

**Figure 1:**
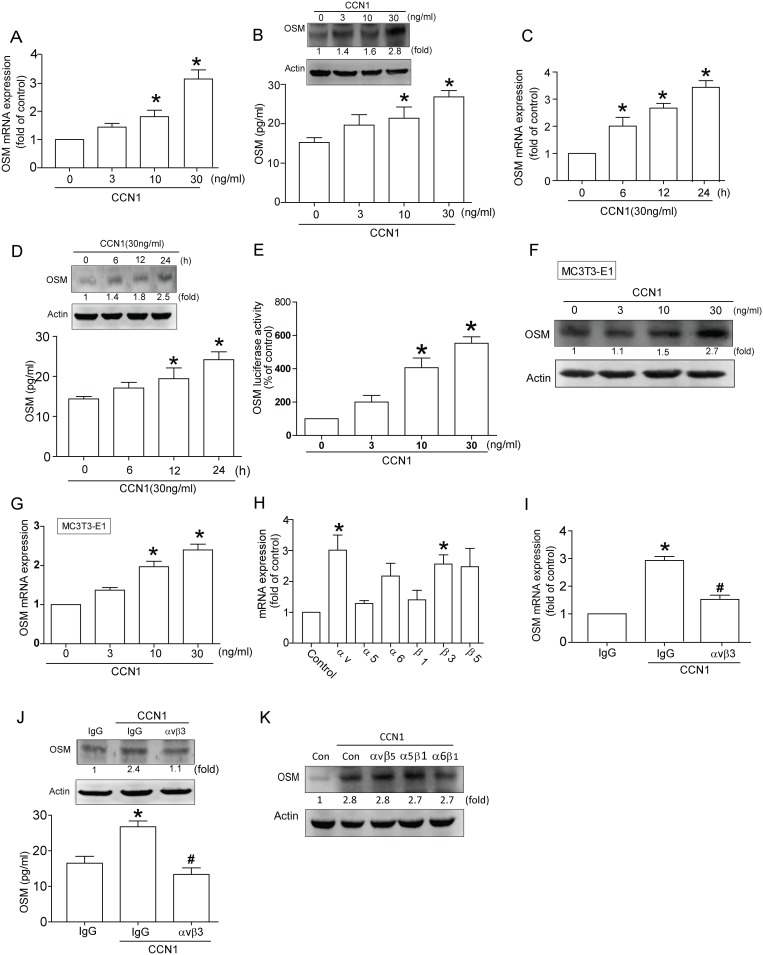
CCN1 enhances OSM expression via αvβ3 integrin in osteoblasts. (A&B) Cells were incubated with various concentrations of CCN1 (3–30 ng/ml), the OSM mRNA and protein levels rated by quantitative polymerase chain reaction (qPCR), western blot, and ELISA respectively. (C&D) Osteoblasts were incubated with CCN1 in time interval, mRNA and protein expression of OSM analyzed by qPCR, western blot, and ELISA, respectively. (E) Osteoblasts were incubated with various concentrations of CCN1 (3–30 ng/ml), the OSM luciferase activity rated by luciferase assay. (F&G) MC3T3-E1 cells were incubated with various concentrations of CCN1 (3–30 ng/ml) for 24, the OSM levels rated by western blot and ELISA. (H) Osteoblasts were incubated with CCN1 for 24 h, mRNA expression of various integrins assessed by qPCR. (I) Cells pretreated with αvβ3 for 30 min were stimulated by CCN1, OSM expression analyzed by qPCR. (J&K) Osteoblasts were pretreated with αvβ3, αvβ5, α5β1 and α6β1 integrin antibody for 30 min followed by stimulation with CCN1 for 24 h, the OSM levels rated by western blot and ELISA. Results are expressed as mean ± S.E. *, p<0.05 compared with control; #, p<0.05 compared with CCN1-treated group.

Earlier study described CCN1 regulating IL-6 expression during arthritis process [4], [20]. Osteoblasts play a vital role in arthritis by producing inflammatory cytokines. We used human osteoblasts to investigate signal pathways of CCN1 in production of OSM (member of IL-6 family). Treatment of osteoblasts with CCN1 (3–30 ng/ml) for 24 h induced OSM mRNA expression in a concentration-dependent manner (Fig. 1A). CCN1 stimulation meant concentration-dependent rise in OSM protein expression, as highlighted by ELISA and Western blot (Fig. 1B&C); this induction occurred in a time-dependent manner (Fig. 1D). On the other hand, CCN1 also increased OSM luciferase activity time-dependently (Fig. 1E). To examine whether OSM expression by CCN1 is a general phenomenon in osteoblasts, the mouse osteoblast cell line MC3T3-E1 was used. CCN1 also increased OSM expression in MC3T3-E1 cells (Fig. 1F&G). Data suggest CCN1 increasing OSM expression in osteoblasts.

### Involvement of αvβ3 receptor in CCN1-induced OSM expression in osteoblasts

Prior studies indicated CCN1 exerting influence through interaction with various integrins [26], [27]; we hypothesized that CCN1 may promote OSM production through integrin receptor. We investigated mRNA expression of OSM with integrins αv, α5, α6, β1, β3, and β5, suggesting αv, α6, β3, and β5 markedly increased after CCN1 treatment; αvβ3 integrin reportedly mediates CCN1-enhanced gene expression [26]. To test the role of αvβ3 integrin in CCN1-induced OSM expression, αvβ3 integrin antibody was used. Incubation of cells with αvβ3 mAb diminished CCN1-increased OSM expression (Figs. 1H–J). However, the other integrin component mAb including αvβ5, α5β1, and α6β1 did not affect CCN1-increased OSM expression (Fig. 1K). These suggest that CCN1-induced OSM expression via integrin αvβ3 receptor in osteoblasts.

### Signaling pathways of FAK and c-Src involved in potentiating action of CCN1

**Figure 2:**
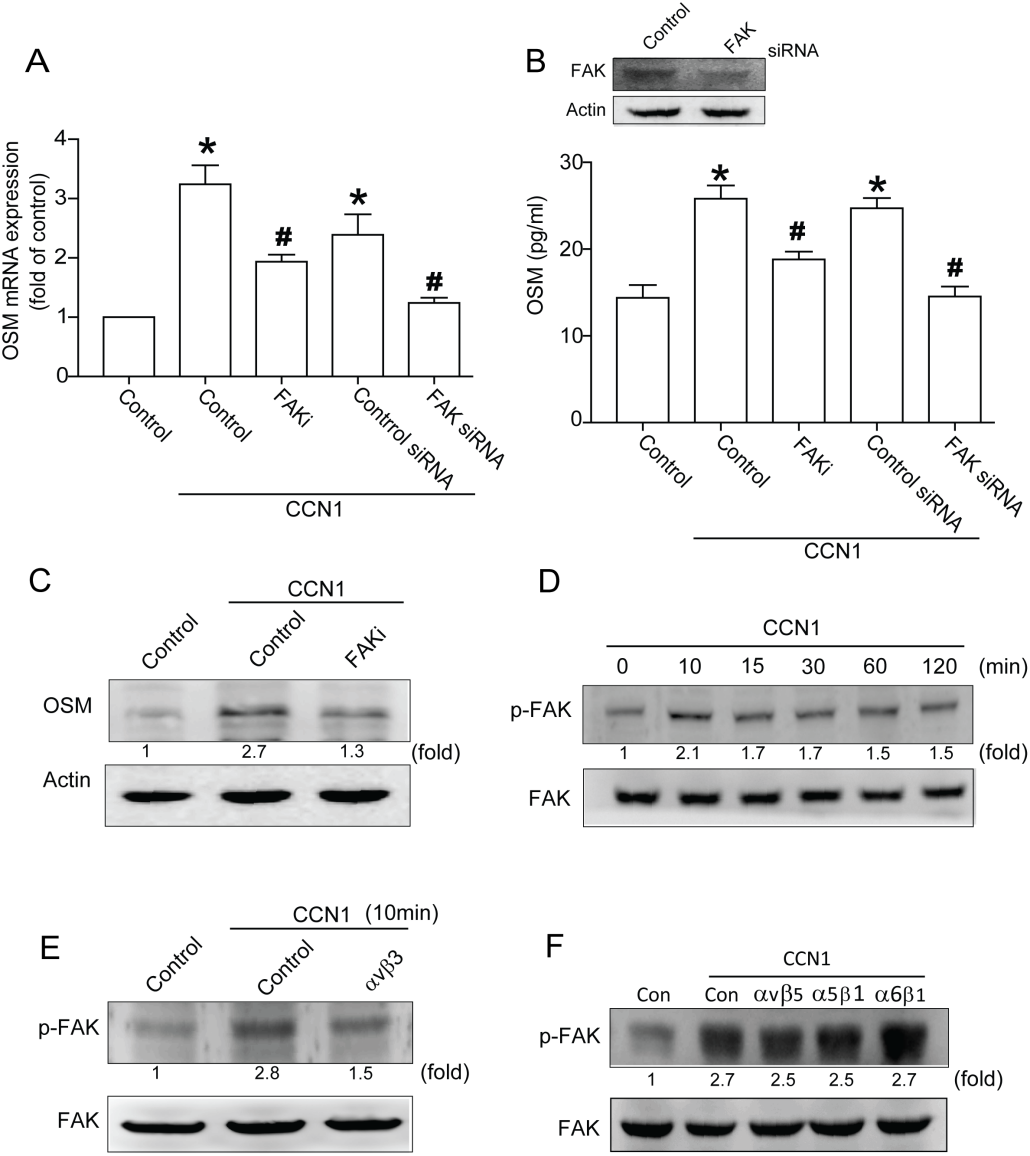
CCN1-induced OSM expression involved FAK signal transduction. (A) Osteoblasts were pretreated with FAK inhibitor (FAKi) (10 µM) or transfected with FAK siRNA (2 µm) for 24 h, then stimulated by CCN1, OSM expression gauged by qPCR. (B) Cells were transfected with FAK siRNA (2 µm) or pretreated with FAKi, protein level of FAK measured by western blot (upper-panel), OSM expression rated by ELISA assay (lower-panel). (C) Cells pretreated with FAKi for 30 min were stimulated with CCN1, protein level of OSM was measured by western blot. (D) Osteoblasts were incubated with CCN1 in time intervals, and p-FAK expression derived by western blot. (E&F) Cells pretreated with αvβ3, αvβ5, α5β1, and α6β1 antibody for 30 min were stimulated with CCN1, p-FAK expression rated by western blot. Data represent mean ± S.E. *, p<0.05 compared with control; #, p<0.05 compared with CCN1-treated group.

**Figure 3:**
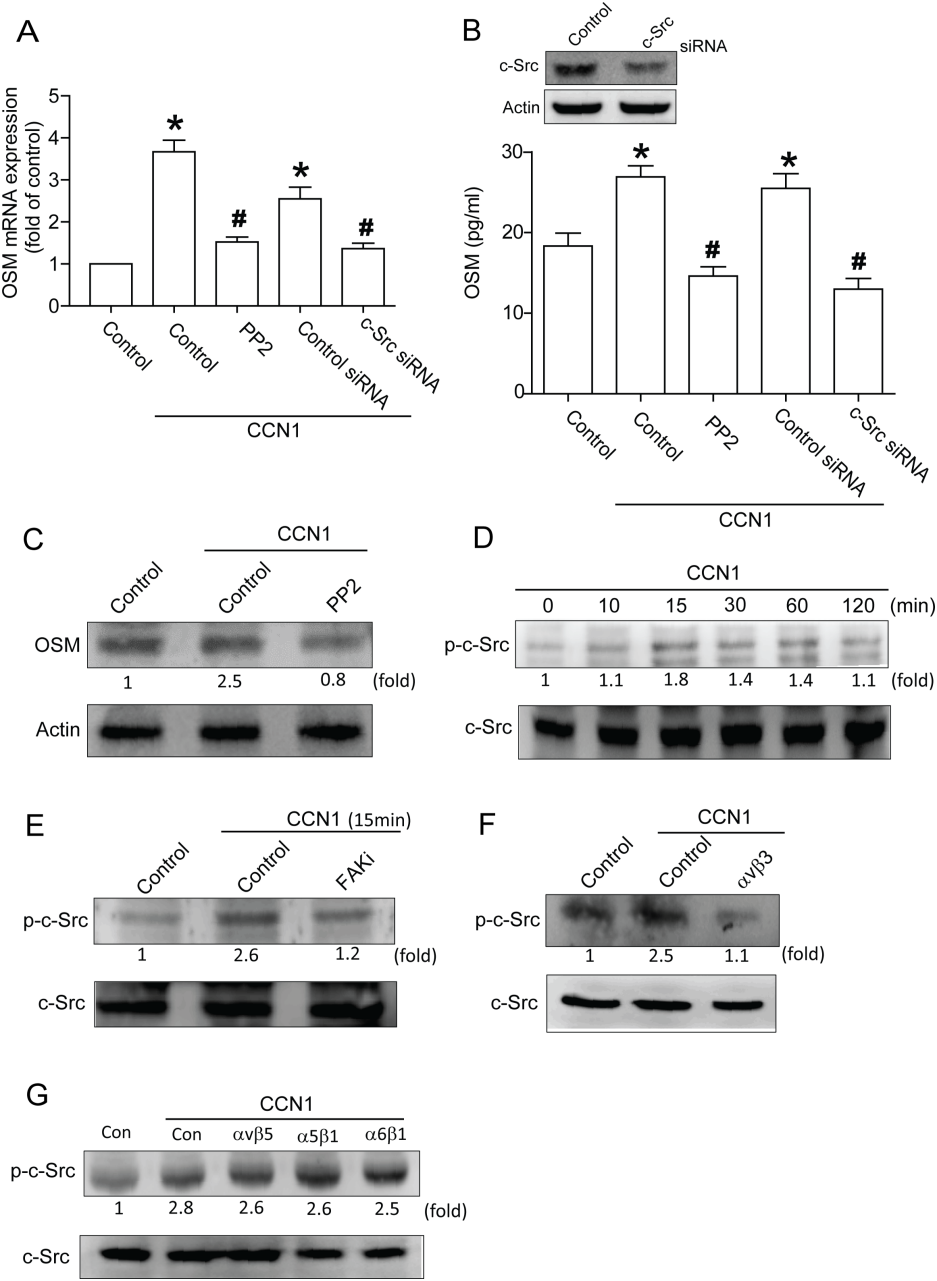
Involvement of c-Src in CCN1-induced OSM expression in osteoblasts. (A) Osteoblasts were pretreated with c-Src inhibitor, PP2, or transfected with FAK siRNA followed by stimulation with CCN1, OSM expression measured by qPCR. (B) Cells were transfected with c-Src siRNA (2 µm) or pretreated with PP2, protein level assessed by western blot (upper-panel), OSM expression measured by ELISA assay (lower-panel). (C) Cells pretreated with PP2 for 30 min follow were stimulated with CCN1, protein level of OSM measured by western blot. (D) Cells were incubated with CCN1 in time intervals, p-c-Src expression rated by western blot. (E) Cells pretreated with FAKi for 30 min were stimulated by CCN1, p-c-Src expression rated by western blot. (F&G) Cells pretreated with αvβ3, αvβ5, α5β1, and α6β1 antibody for 30 min were stimulated with CCN1, p-c-Src expression rated by western blot. Data represent mean ± S.E. *, p<0.05 compared to control; #, p<0.05 compared with CCN1-treated group.

FAK purportedly regulates integrin-mediated signal in cell function [28], [29]. To verify its involvement in CCN1-mediated OSM expression, cells were pretreated with FAK inhibitor or transfected with FAK siRNA; both abolished CCN1-induced OSM production (Fig. 2A–C). Next, phosphorylation of FAK in osteoblasts after CCN1 stimulation was tabulated. Treatment with CCN1 promoted Tyr^397^ phosphorylation of FAK in a time-dependent manner (Fig. 2D). Conversely, αvβ3 but not αvβ5, α5β1 or α6β1 antibody pretreatment decreased CCN1-induced FAK phosphorylation (Fig. 2E&F), suggesting CCN1-induced OSM expression via integrin αvβ3 and FAK signal pathways. Previous study showed FAK-dependent c-Src activation crucial to inflammatory cytokine production [30]. We examined whether c-Src is a downstream effector of FAK. Figures 3A–C show the mRNA and protein of OSM reduced by c-Src inhibitor PP2 pretreatment or c-Src siRNA transfection. To ascertain whether CCN1 activated c-Src, we used antibody against c-Src to prove activation of c-Src. Stimulation with CCN1 enhanced c-Src phosphorylation time-dependently (Fig. 3D); FAK inhibitor pretreatment abolished CCN1-promoted c-Src phosphorylation (Fig. 3E). In addition, pretreatment with αvβ3 but not αvβ5, α5β1 or α6β1 antibody reduced CCN1-incrased c-Src phosphorylation (Fig. 3F&G). These indicate CCN1 increased OSM expression through αvβ3 receptor and activated FAK-dependent c-Src pathway.

### Involvement of PI3K in CCN1-induced OSM expression in osteoblasts

**Figure 4:**
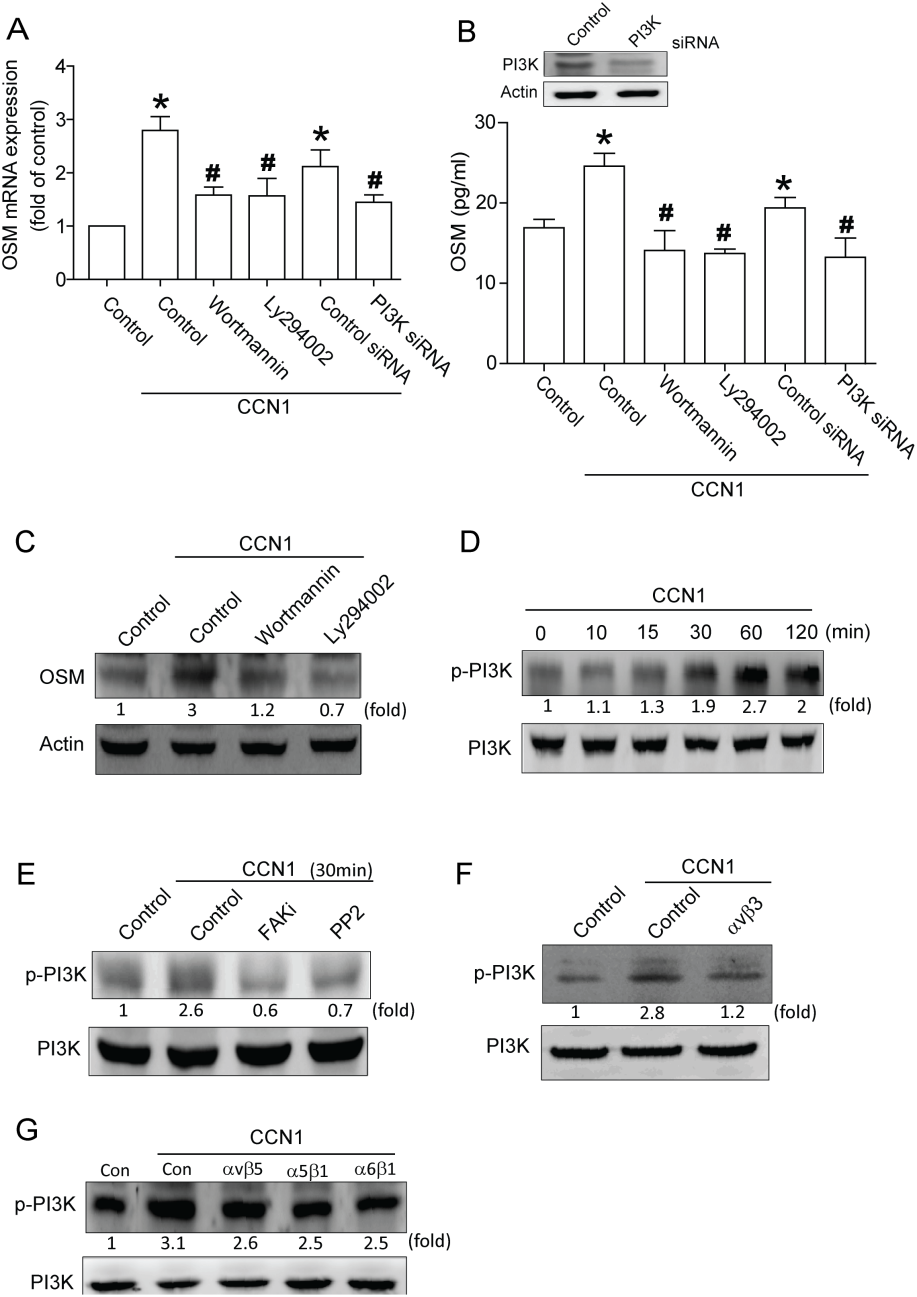
Role of PI3K in CCN1-mediated OSM expression. (A) Osteoblasts were pretreated with Wortmannin (10 µM) and Ly294002 (10 µM) or transfected with PI3K siRNA (2 µm) before stimulation with CCN1, mRNA expression of OSM analyzed by qPCR. (B) Cells were transfected with PI3K siRNA or pretreated with Wortmannin and Ly294002, protein level measured by western blot (upper-panel), OSM expression by ELISA assay (lower-panel). (C) Cells pretreated with Wortmannin and Ly294002 for 30 min were stimulated with CCN1, OSM protein level measured by western blot. (D) Cells were incubated with CCN1 in time intervals, p-PI3K expression gauged by western blot. (E) Cells pretreated with FAKi or PP2 for 30 min were stimulated by CCN1, p-PI3K expression rated by western blot. (F&G) Cells pretreated with αvβ3, αvβ5, α5β1, and α6β1 antibody for 30 min were stimulated with CCN1, p-PI3K expression rated by western blot. Data represent mean ± S.E. *, p<0.05 compared with control; #, p<0.05 compared with CCN1-treated group.

PI3K activation reportedly takes part in OSM production [31], [32]. We continued to investigate PI3K involvement in CCN1-induced OSM production. Pretreatment with PI3K inhibitors Wortmannin and Ly294002 or transfection with PI3K siRNA reduced CCN1-increased OSM expression (Fig. 4A–C). PI3K phosphorylation increased in a time-dependent manner in response to CCN1 stimulation. (Fig. 4D); CCN1-induced PI3K phosphorylation was markedly inhibited in cells pretreated for 30 min with FAK inhibitor or PP2 (Fig. 4E). On the other hand, αvβ3 but not αvβ5, α5β1 or α6β1 antibody reduced CCN1-incrased PI3K phosphorylation (Fig. 4F&G). Results suggest CCN1-induced OSM expression mediated through FAK/c-Src/PI3K pathway.

### CCN1 increases OSM expression through NF-κB pathway

**Figure 5:**
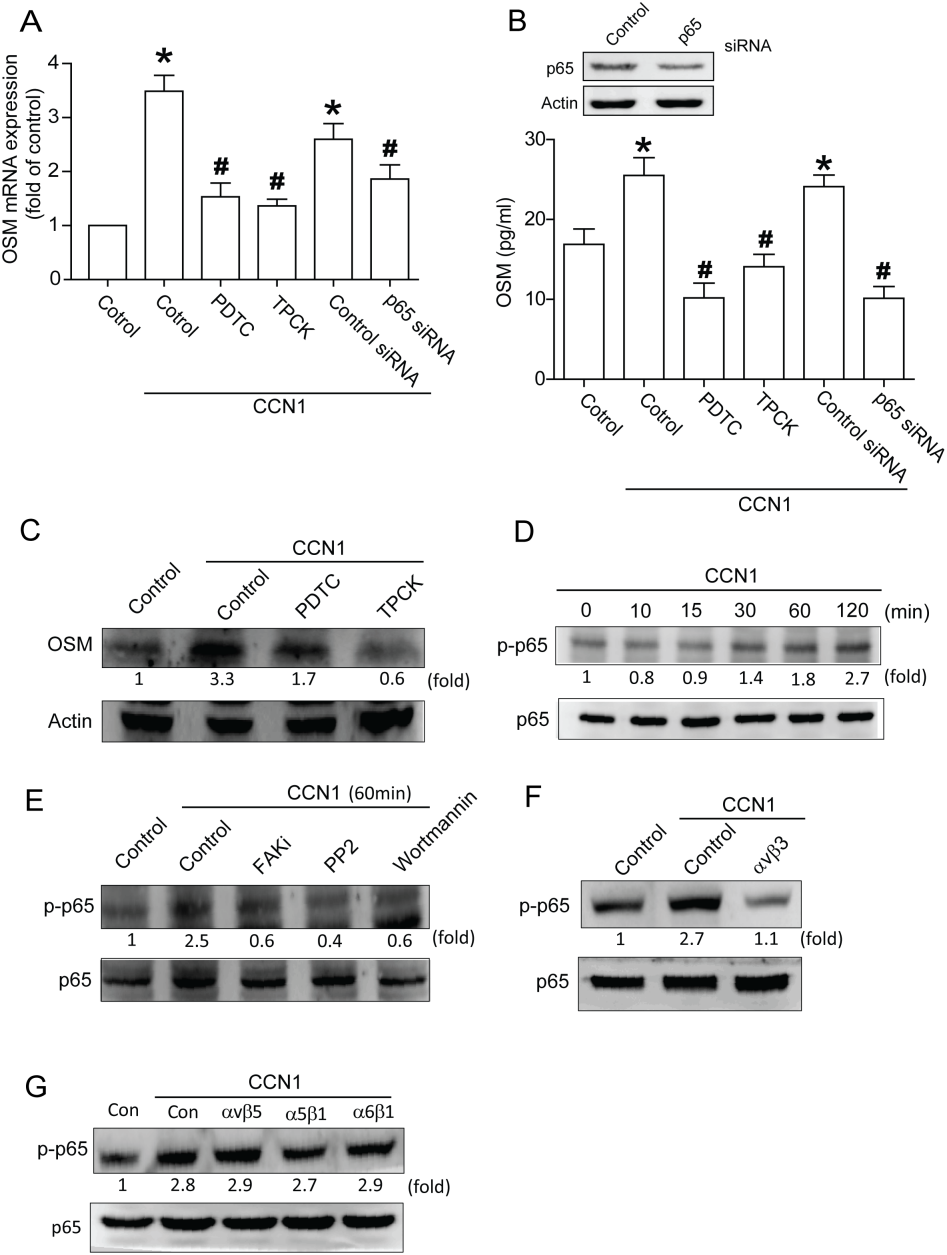
NF-κB is involved in CCN1-induced OSM production in osteoblasts. (A) Osteoblasts were pretreated with PDTC (10 µM) and TPCK (10 µM) or transfected with p65 siRNA (2 µm) followed by stimulation with CCN1, mRNA expression of OSM analyzed by qPCR. (B) Cells were transfected with p65 siRNA or pretreated with PDTC and TPCK, the protein level was measured by western blot (upper-panel), and OSM expression was measured by ELISA assay (lower-panel). (C) Cells were pretreated with PDTC and TPCK for 30 min followed by stimulation with CCN1, the protein level of OSM was measured by western blot. (D) Cells were incubated with CCN1 in time intervals, p-p65 expression evaluated by western blot. (E) Cells were pretreated with FAKi, PP2, Wortmannin or Ly294002 for 30 min followed by stimulation with CCN1, p-p65 expression was investigated by western blot. (F&G) Cells pretreated with αvβ3, αvβ5, α5β1, and α6β1 antibody for 30 min were stimulated with CCN1, p-p65 expression rated by western blot. Results are expressed as mean ± S.E. *, p<0.05 compared with control; #, p<0.05 compared with CCN1-treated group.

NF-κB, a transcription factor, plays a crucial role in immune and inflammatory responses [33], [34]. To learn whether NF-κB activation is involved in CCN1-mediated OSM expression, NF-κB inhibitors PDTC and TPCK were used. Pretreatment with PDTC or TPCK reduced CCN1-enhanced OSM expression (Fig. 5A–C). Transfection with p65 siRNA abolished CCN1-increased OSM expression (Fig. 5B). On the other hand, stimulation of osteoblasts with CCN1 increased p65 phosphorylation in time dependent manner (Fig. 5D). Furthermore, pretreatment of cells with FAK inhibitor, PP2, or Wortmannin inhibited the CCN1-promoted p65 phosphorylation (Fig. 5E). Incubation of cells with αvβ3 but not αvβ5, α5β1 or α6β1 antibody abolished CCN1-incrased p65 phosphorylation (Fig. 5F&G). Results indicate activation of NF-κB critical to CCN1-mediated OSM expression.

**Figure 6:**
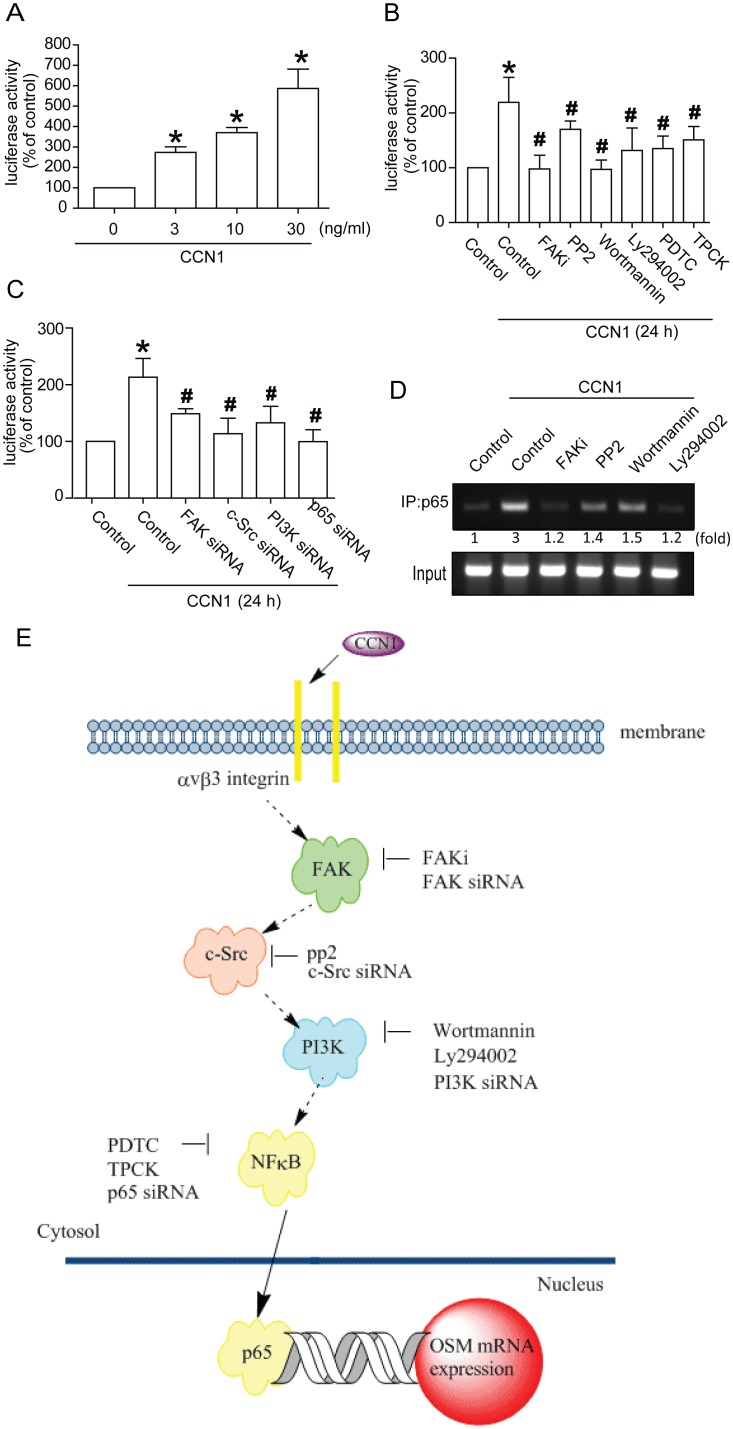
Activation of NF-κB enhances CCN1-induced OSM production. (A) Osteoblasts were incubated with various concentrations of CCN1 (3–30 ng/ml) in NF-κB luciferase activity. (B&C) Osteoblasts pretreated with FAK inhibitor, PP2, Wortmannin, Ly294002, PDTC, and TPCK for 30 min or transfected with FAK, c-Src, PI3K, and p65 siRNA were treated with CCN1. NF-κB luciferase activity measured, results normalized to β-galactosidase activity and expressed for three independent experiments performed in triplicate. (D) Osteoblasts pretreated with FAKi, PP2, Wortmannin, LY294002 for 30 min were stimulated with CCN1 for 60 min, followed by chromatin immunoprecipitation assay. (E) Schematic diagram of signal pathway showed CCN1-induced OSM expression in osteoblasts. Data represent mean ± S.E. *, p<0.05 compared with control; #, p<0.05 compared with CCN1-treated group.

NF-κB activation was further analyzed with κB-luciferase activity and chromatin immunoprecipitation assay. To determine NF-κB activation after CCN1 treatment, osteoblasts were transiently transfected with NF-κB luciferase reporter as an indicator of NF-κB activation. We found 24 h CCN1 treatment boosting this luciferase activity in a concentration-dependent manner (Fig. 6A). Pretreatment with FAK inhibitor, PP2, Wortmannin, Ly294002, PDTC, and TPCK inhibited CCN1-induced NF-κB luciferase activity (Fig. 6B). Co-transfection of cells with siRNA against FAK, c-Src, PI3K, and p65 also reduced CCN1-enhanced NF-κB luciferase activity (Fig. 6C). We next probed p65 binding to NF-κB element on OSM promoter after CCN1 stimulation; *in* *vivo* binding of p65 to NF-κB element of OSM promoter occurred after CCN1 stimulation (Fig. 6D). Binding of p65 to NF-κB element by CCN1 was attenuated by FAK inhibitor, PP2, Wortmannin, and Ly294002 (Fig. 6D). Activation of the FAK/c-Src/PI3K/NF-κB pathway is needed to raise CCN1-induced OSM expression.

## Discussion

OA, also called degenerative arthritis, is one common symptomatic disease. Progressive degradation of articular cartilage leads to joint dysfunction, disability, and inflammation. [35]. OA, a symmetric polyarthritis, impairs joints–e.g., meta-carpaphalangeal, proximal, interphalangeal–resulting in cartilage destruction [36]. Since OSM is constitutively expressed in the bone compartment and detected in patients with arthritis pathology [23], using OSM antibody could decrease cartilage destruction of knee joints *in* *vivo* [37]. This study identified OSM as a target protein for regulation of cell inflammatory response. In the current study, the basal OSM concentration in culture medium is approximate 15 pg/ml. The OSM mRNA and protein expression in cell lysates was somewhat different from it in the culture medium. It’s meaning the basal OSM concentration in culture medium is not very higher. Therefore, the OSM is difficult to secret into medium but located in cytosol in MG-63 cells. Whether other osteoblast cell lines have same effect are need further examination. We also showed potentiation of OSM activated by CCN1 via FAK/c-Src/PI3K/NF-κB signal pathways in osteoblasts.

Previous study correlated arthritis with heparin-binding protein, CCN1 [38], [39]. CCN1 has been shown to stimulate abnormal proliferation and play a critical role in arthritis pathogenesis [39]. It also regulates inflammatory cytokines, osteogenesis, and osteoblasts differentiation through αvβ3/ILK signal pathway [18] and thus might hold potential as therapy through mTORC2/Akt/IκB/NF-κB signal pathway in human osteoblasts [40]. Recent study shows osteoblasts that secrete inflammatory mediators involved in arthritic process [6]; CCN1-regulated OSM behavior and mechanisms remain unclear. First, we identified CCN1 signal pathways in production of OSM by using human osteoblasts, suggesting CCN1 as progressing novel treatment of OA.

Prior studies indicated CCN1-activated integrin receptor applying to arthritis [41], [42]: binding of CCN1 to αvβ5 or α6β1 could promote cell apoptosis and reactive oxygen species generation [43]. Our results prove αvβ3 integrin involvement in CCN1-induced OSM expression, with pretreatment of αvβ3 antibody decreasing CCN1-induced OSM production. Recent studies show blocking relevant integrins can avoid leukocyte infiltration and inhibit pathological inflammation [44]. Therefore, αvβ3 integrin might play an important role in osteoblasts of OA inflammation.

FAK, common signal protein, participates in numerous vital mechanisms [28]. Ever more reports show activation of FAK requiring αvβ3 integrin that plays a lead role in this mechanism [45]. Previous studies also showed interaction of CCN1 and FAK mediating cell apoptosis in fibroblasts [46] while inducing cell migration and angiogenesis by MMP family [47], [48]. Our early study highlighted CCN1 activated MMP-3 expression and cell migration through αvβ3 receptor and FAK/ERK/AP-1 in human chondrosarcoma [49]. In this study, neither pretreating inhibitor of FAK nor transfection with FAK siRNA would impair CCN1-induced OSM expression in osteoblasts. Continuously, previous study has shown degradation of ECM linked with FAK/c-Src signal pathway [50] and c-Src correlating with inflammation [51], but interaction between CCN1 and c-Src was rare in previous study. Yet CTGF-mediated FAK/c-Src signal pathway reportedly suppresses chondrogenesis in early stages of bone remodeling [30]. We found FAK inhibitor and c-Src pretreatment or transfection with c-Src siRNA potently abolishing CCN1-mediated OSM expression, portending FAK-dependent c-Src signal pathway involved in CCN1-induced OSM expression. FAK-dependent c-Src activation is implicated in cell motility, cell cycle, or inhibited neuroblastoma progression [28], [52]. Studies showed this pathway regulating human epithelial cell survival and anoikis [53]. Together, FAK/c-Src signal pathway might cooperate in inflammation response.

NF-κB is a common transcriptional factor in numerous signal pathways, due to its regulatory inflammation. Its activation could regulate gene transcription and encode inflammatory proteins involved in immune responses [54], [55]. Arthritis is a chronic inflammatory disease, so NF-κB may be therapeutic strategy [57]. Recent study shows CCN1 inducing inflammatory cytokine CCL2 through FAK, PI3K/Akt, and NF-κB pathways in retinal vascular endothelial cells [56]. These studies indicated NF-κB activation as critical to inflammation. Our data showed CCN1-induced OSM expression via αvβ3 receptor/FAK/c-Src/PI3K, followed by NF-κB transcriptional factor in osteoblasts. Both NF-κB inhibitor and p65 siRNA effectively impaired CCN1-induced OSM expression. As for binding of OSM, AP-1 is discussed widely, that of OSM and NF-κB rarely [57]. Findings highlight how CCN1 augments binding of NF-κB element on OSM promoter, as shown by chromatin immunoprecipitation assay, suggesting NF-κB as chief binding site for CCN1-induced OSM production, binding of NF-κB element attenuated by FAKi, PP2, Wortmannin, Ly294002. We transfected with κB luciferase as an indicator of κB activity to note CCN1 enhancing κB activity, which was impaired by upstream inhibitors of FAKi, PP2, Wortmannin, Ly294002. These indicate CCN1 acting through αvβ3, FAK, c-Src, PI3K, NF-κB signal pathways to produce OSM in human osteoblasts. In sum, we explored signal pathways to find CCN1 upgrading OSM expression by binding to αvβ3 receptor and activating FAK/c-Src/PI3K/NF-κB signal pathways in osteoblasts. Results helped us understand mechanisms whereby CCN1 produces OSM underlying inflammatory response and has potential for OA therapy.
